# Mitochondrial Variants in Pompe Disease: A Comparison between Classic and Non-Classic Forms

**DOI:** 10.22074/cellj.2018.5238

**Published:** 2018-05-28

**Authors:** Fatemeh Bahreini, Massoud Houshmand, Mohammad Hossein Modarressi, Seyed Mohammad Akrami

**Affiliations:** 1 Department of Medical Genetics, School of Medicine, Tehran University of Medical Sciences, Tehran, Iran; 2 Department of Molecular Medicine and Genetics, School of Medicine, Hamadan University of Medical Sciences, Hamadan, Iran; 3 Department of Medical Genetics, National Institute of Genetic Engineering and Biotechnology, Tehran, Iran

**Keywords:** Alpha-Glucosidase, Cytochrome-C Oxidase, Mitochondria, Pompe

## Abstract

**Objective:**

Pompe disease (PD) is a progressive neuromuscular disorder that is caused by glucosidase acid alpha (GAA) deleterious mutations. Mitochondrial involvement is an important contributor to neuromuscular diseases. In this study the sequence of *MT-ATP 6/8* and Cytochrome C oxidase *I/II* genes along with the expression levels of the former genes were compared in classic and non-classic patients.

**Materials and Methods:**

In this case-control study, the sequence of *MT-ATP 6/8* and Cytochrome C oxidase was analyzed by polymerase chain reaction (PCR)-Sanger sequencing and expression of *MT-ATP* genes were quantified by real time-PCR (RT-PCR) in 28 Pompe patients. The results were then compared with 100 controls. All sequences were compared with the revised Cambridge reference sequence as reference.

**Results:**

Screening of *MT-ATP6/8* resulted in the identification of three novel variants, namely T9117A, A8456C and A8524C. There was a significant decrease in *MT-ATP6* expression between classic (i.e. adult) and control groups (P=0.030). Additionally, the MT-ATP8 expression was significantly decreased in classic (P=0.004) and non-classic (i.e. infant) patients (P=0.013). In total, 22 variants were observed in *Cytochrome C oxidase*, five of which were non- synonymous, one leading to a stop codon and another (C9227G) being a novel heteroplasmic variant. The A8302G in the lysine tRNA gene was found in two brothers in a pedigree, while a T7572C variant in the aspartate tRNA gene was observed in two brothers in another pedigree.

**Conclusion:**

The extent of mitochondrial involvement in the classic group was more significant than in the non-classic form. Beside *GAA* deleterious mutations, it seems that mtDNA variants have a secondary effect on PD. Understanding, the role of mitochondria in the pathogenesis of Pompe may potentially be helpful in developing new therapeutic strategies.

## Introduction

Pompe disease (PD, OMIM #232300) is a progressive myopathy with an autosomal recessive mode of inheritance (1). The combined incidence of PD is generally 1 in 40,000 (2). It has two common forms (early-onset/classic and late-onset/non-classic) with differences in degree of disease severity, age of onset and organ involvement (3, 4). The patients present a broad spectrum of clinical variability such as cardiomyopathy, hypotonia and respiratory insufficiency (5, 6).

They suffer from deficiency or lack of acid alphaglucosidase enzyme (*GAA*) that arise as a result of various deleterious variants in *GAA* (1). Genotype-phenotype correlation studies among patients with the same mutation in *GAA* have revealed different clinical manifestations (2). It seems that this diversity may be a result of interaction of other genetic and non-genetic factors. The sign and symptoms that are observed in Pompe patients are similar to those in mitochondrial disorders.

According to previous reports, mitochondrial dysfunction can affect the neuromuscular system (7). Mitochondria (mt) are essential to aerobic respiration by producing adenosine triphosphate (ATP). The function of mt is controlled by both the mtDNA and nuclear genomes, and mtDNA variants may be affected by nuclear genome variants or vice versa (8). It is therefore possible that mtDNA genes interact with GAA. To test this hypothesis, *MT-ATP6/8* and *Cytochrome C oxidase I/II* were screened for functional variants, and the expression level of the former genes were analyzed in early and late- onset PD patients.

## Materials and Methods

In this case-control study, we recruited 28 PD patients (17 infants and 11 adults) from the Department of Neurology of both Shariati and Mofid hospitals from December 2013 to February 2015. In this study, 100 healthy controls were also recruited comprising 17 infants and 83 adults. An informed consent was obtained from each participant or a parent in the case of infants. PD was diagnosed based on clinical findings by two expert neurologists, measurement of *GAA* biochemical activity or detection of deleterious variants in *GAA*. The included patients had no family history of mitochondrial or major neuromuscular disorders. This study was approved by the Ethical Committee of Tehran University of Medical Sciences (92-02-30-23162). 

## DNA/RNA extraction 

DNA was extracted from whole blood by using QIAamp DNA Blood Mini Kit (QIAGEN, Germany) according to the manufacturer’s instructions. Quantity and quality of DNA were checked by NanoDrop ND-1000 (NanoDrop Technologies, USA) at 260/280 nm wavelengths and running on an agarose gel (1%) respectively. Total RNAwas extracted from fresh whole blood samples by using the Hybrid-RTM Blood RNA kit and following its protocol (see http://www. tribioscience.com/files/315-150.pdf). RNA concentration and integrity were measured by NanoDrop and agarose gel respectively prior to cDNA synthesis. Presence of sharp bands for both 18S and 28S rRNA was checked. Purified RNA was then stored at -80°C.

## cDNA synthesis

Total RNA was used to synthesize cDNA by using the cDNA synthesis kit (Fermentas, Germany) according to manufacturer’s instructions. Briefly, 2 µg of total RNA, 1 µL of oligo dT and random hexamer primers and 8 µL nuclease-free water were mixed in a sterile, nuclease- free tube and placed on ice. After incubation at 65°C for 5 minutes, it was chilled on ice and 4 µL of 5X reaction buffer, 1 µL of RiboLock RNase Inhibitor (20 U/µL), 2 µL of 10 mM dNTP mix, 1 µL of RevertAid M-MuLV RT (200 U/µL) were added. 

The mixture was centrifuged briefly and incubated for 5 minutes at 25°C followed by 60 minutes at 42°C. The reaction terminated by heating at 70°C for 5 minutes and stored at -80°C until further use.

## Variant detection 

Polymerase chain reaction (PCR) was performed with primers specific to *MT- ATP6/8, Cytochrome C* oxidase and their flanking sequences (Table 1) (8). 

The PCR reaction included 50 ng of genomic DNA, 1 µL of each primer (10 pmol), 0.2 mM of each deoxynucleoside triphosphate (dNTP), 1.5 mM MgCl_2_ and 1 U of Taq polymerase (CinnaGen, Inc, Iran). Cycling conditions for all PCR reactions were an initial denaturation at 94°C for 4 minutes, followed by 35 cycles of denaturation at 94°C for 35 seconds, annealing at 56° for 35 seconds, extension at 72°C for 35 seconds, and a final extension at 72°C for 5 minutes. PCR-amplified fragments were sequenced by Macrogen (South Korea) using the same PCR primers in both directions along with a series of overlapping primers to cover all regions of interest for more accurate results. Finch TV version 1.4 (Geospiza, Inc., USA) was used to analyze the chromatograms and were then checked using BLAST (https://blast.ncbi.nlm.nih.gov). The results were compared with the revised Cambridge reference sequence MITOMAP (www.mitomap.org) and the 1000 Genome databases. Presence of variants was also checked in controls which were selected from different Iranian ethnicities. Furthermore, the effect of missense variants on protein structure was assessed by Polyphen-2 (http://genetics.bwh.harvard.edu/pph2) and CADD (Combined Annotation Depedent Depletion; http:// cadd.gs.washington.edu/score) scores. 

## *MT-ATP* 6/8 expression analysis

Expression levels of *MT-ATP 6/8* were quantified using a quantitative PCR (qPCR) assay. In brief, expression values were normalized relative to a housekeeping gene (*ß-actin*) to calculate the relative gene expression based on the 2^-ΔΔCt^ method (9). Details of primers are given in Table 2.

qPCR reactions were performed on a Corbett 6000 PCR-Real-time Detection System with a total volume of 20 µl reaction mixture, containing 1 µl DNA template (50 ng), 10 µl SYBR Green PCR Master Mix (Takara, Japan), 8 µl nuclease- free water and 0.5 µl of each primer (10 pmol). The cycling conditions were an initial denaturation step at 95ºC for 30 seconds followed by 40 cycles of a denaturation step at 95ºC for 12 seconds and an annealing step at 58ºC for 35 seconds. Melting curve analysis was used to validate the specificity and identity of the PCR product for each primer pair. Each sample was run in duplicate to ensure the reliability of the results. Also, a non-template control was included in each qPCR run. 

## Statistical analysis 

Quantitative variables, in the form of frequency, such as participant characteristics and mitochondrial involvement were described as mean ± SD. Fisher’s exact test was used to compare frequency of mitochondrial involvement in PD and control groups. P<0.05 was considered statistically significant. All analyses were implemented in SPSS version 16 (IBM, USA). Bonferroni’s multiple-testing correction was used to adjust the significance level (a). For frequency comparison of the identified 14 variants, a was set to 0.0036. For differential expression, given that two genes were compared, a was set to 0.025. 

## Results

**Fig.1:**
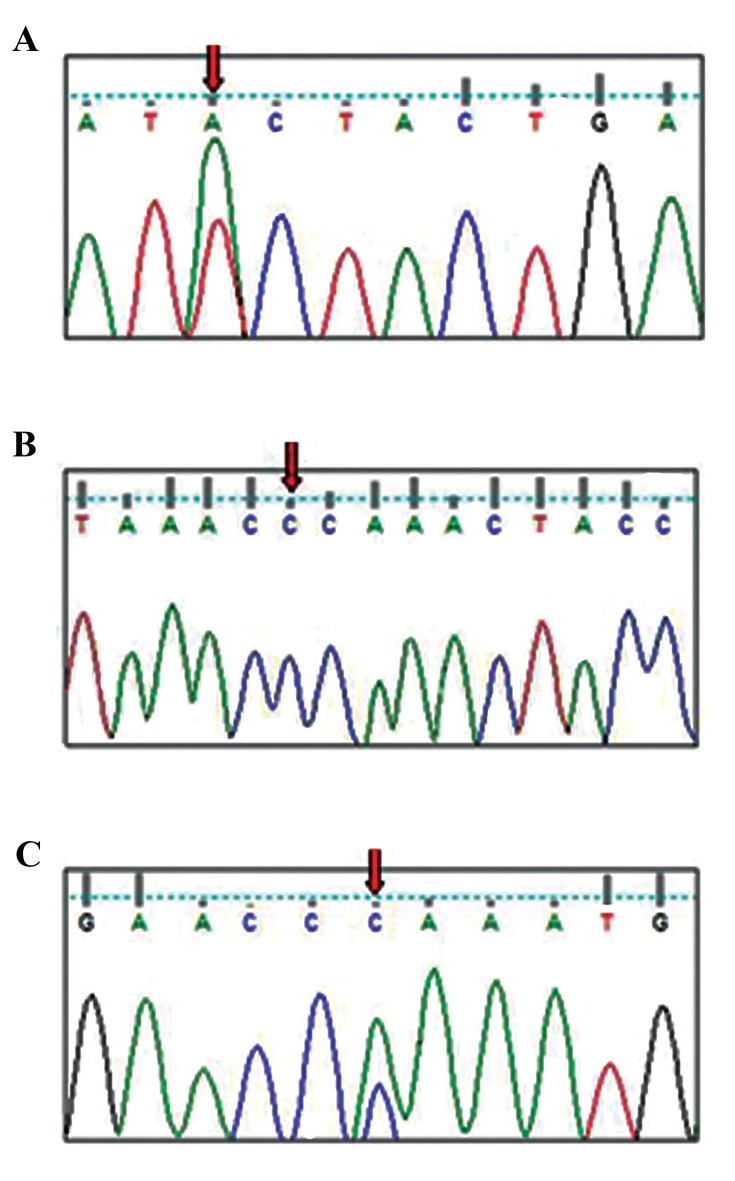
Chromatogram of the three novel variants in Mt.ATPase6/8 genes. A. T9117A, p.I197I, Heteroplasmy, B. A8456C, p.T31P, Homoplasmy, and C. A8524C, p.P53P, Heteroplasmy.

Screening of MT-ATP6/8 in patients resulted in the identification of 14 variants, of which three were novel variants (Fig .1). Of the total, 7 variants were in the classic group, 4 in the non-classic group and 3 were shared between the two groups (Table 1). There were four synonymous and ten non synonymous variants in *MT-ATPase6/8* (Table 1). The variants not previously reported in MITOMAP (WWW.mitomap.org) and other variant databases (e.g. the 1000 Genome database), were checked in controls. Frequencies of A8524C and C8562T were significantly different between patients and controls (P=0.047). A8860G, A8524C and C8562T were observed in 85.71, 7.14 and 7.14% of patients, respectively. The Polyphen-2 and CADD scores of missense variants showed in the Table 2. 

**Table 1:** Comparison of MT-ATP6/8 gene variants in PD and control groups


Infant/Adult	Nucleotide	Locus	Amino acid change	R/N.R	Hm/Ht	Pompe	Control	P value
						+	+	

Adult	C8406T	MT-ATPase8	p.T14I	R	Hm	1	0	0.219
	A8456C	MT-ATPase6/8	p.T31P	N.R	Hm	1	0	0.219
	G9039A	MT-ATPase6	p.M171I	R	Hm	1	0	0.219
	G9055A	MT-ATPase6	p.A177T	R	Hm	1	3	0.99
Infant	A8502T	MT-ATPase8	p.N46I	R	Hm	1	0	0.219
	C8562T	MT-ATPase6	p.P66L	R	Hm	2	0	0.047
	C8562T	MT-ATPase8	p.P66P	R	Hm	2	0	0.047
	C8684T	MT-ATPase6	p.T53I	R	Hm	1	6	0.650
	G8697A	MT-ATPase6	p.M57I	R	Hm	1	0	0.219
	T9117A	MT-ATPase6	p.I197I	N.R	Ht	1	0	0.219
	C9129T	MT-ATPase6	p.I201I	R	Hm	1	0	0.219
Both	A8524C	MT-ATPase8	p.P53P	N.R	Ht	2	0	0.047
	A8701G	MT-ATPase6	p.T59A	R	Hm	2	6	0.556
	A8860G	MT-ATPase6	p.T112A	R	Hm	24	81	0.235


R and N.R; Denote reported and not-reported respectively, Hm and Ht; Denote homoplasmy and heteroplasmy respectively, and PD; Pompe disease.

**Table 2:** Predicted effect of missense variants on protein structure


Gene	Nucleotide position	Polyphen-2 score	CADD score	Prediction effect

MT-CO2	7805	0.00	0.01	Benign
MT-CO2	7859	0.00	0.50	Benign
MT-ATP8	8406	0.08	0.16	Benign
MT-ATP8	8456	0.02	3.82	Benign
MT-ATP8	8502	0.99	18.47	Damaging
MT-ATP6	8701	0.00	0.09	Benign
MT-ATP6	9039	0.89	18.04	Damaging
MT-ATP6	9055	0.84	22.60	Damaging
MT-CO3	9336	0.00	0.01	Benign
MT-CO3	9949	0.99	23.60	Damaging
MT-CO3	9963	1.00	23.70	Damaging


## *MT-ATP6/8* genes expression

A significant decrease was observed for *MT-ATP6* expression inclassic group compared with the control group. In addition, *MT-ATP8* expression was significantly decreased in the classic (P=0.004) and non-classic (P=0.013) patient groups compared with their controls.

## *MT-Cytochrome C* oxidase

**Fig.2:**
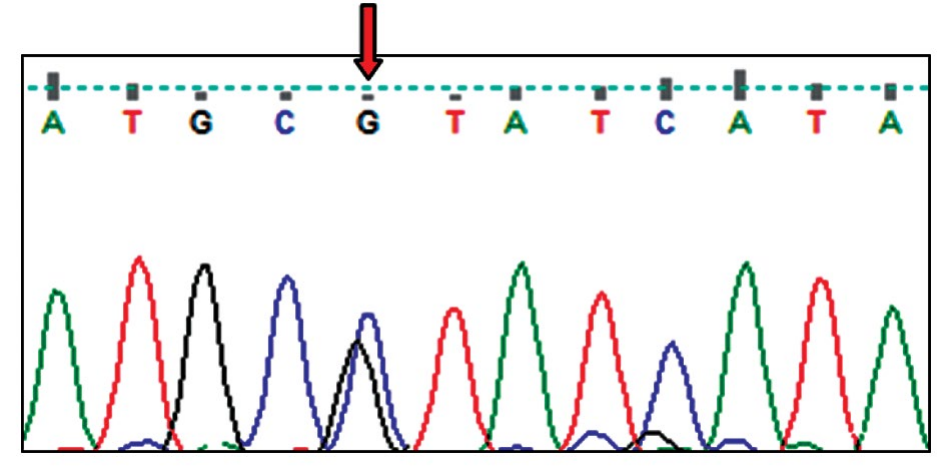
Chromatogram of *C9227G* as a novel heteroplasmic variant in the *Mt. Cytochrome C oxidase* gene.

Screening of MT-Cytochrome *C oxidase* led to the observation of 22 variants which C9227G sequence being a new heteroplasmic variant (Fig .2). Fifteen and five variants were identified in the classic and non-classic groups respectively, while two variants were shared between the two groups. Furthermore, 5 variants were non-synonymous, 15 synonymous with one stop codon (Table 3). The predicted effect of missense variants on protein structure are given in Table 2.

**Fig.3:**
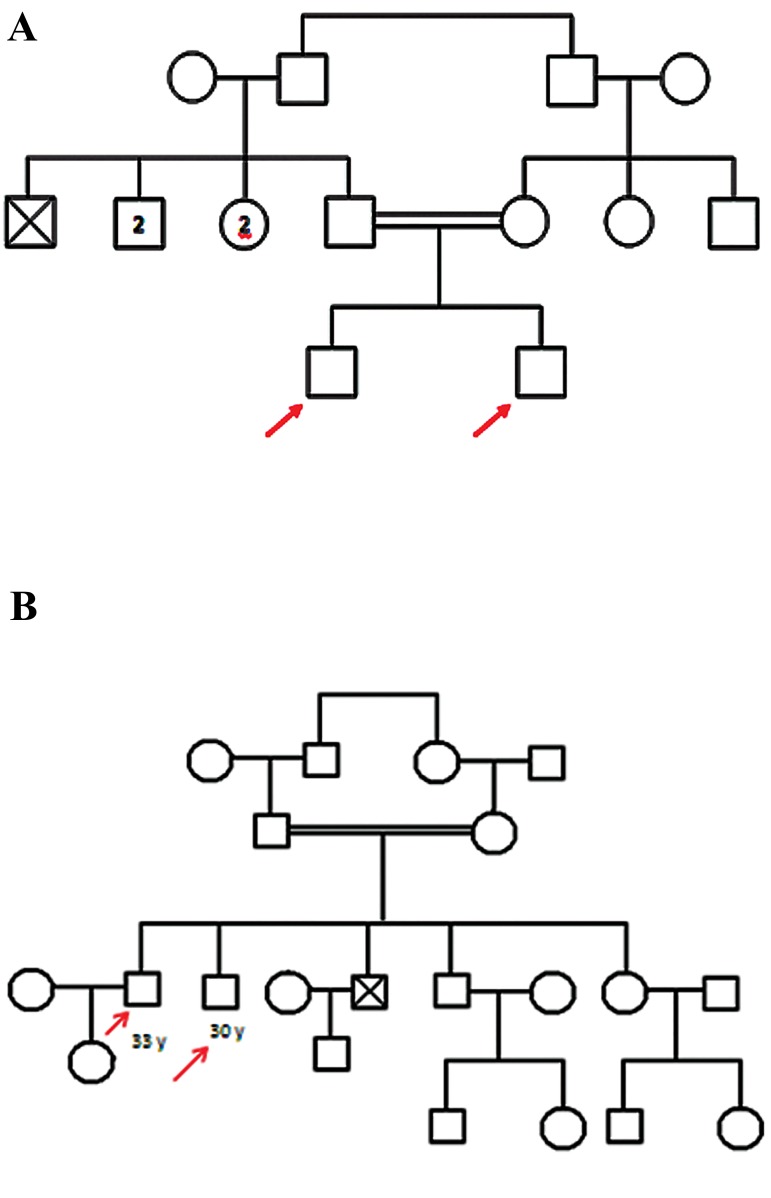
Pedigree diagram. A. Pedigree of a family with a A8302G variant in siblings and B. Pedigree of a family with a T7572C variant in siblings.

Moreover, based on the analysis of the sequence of lysine tRNA and aspartate tRNA genes, the A8302G variant was found in the former gene in two brothers in a pedigree (Fig .3A) and the T7572C variant was found in the latter gene in two brothers in another pedigree (Fig .3B). Variants C15904T and G15928A were also found in two infants in the threonine and tyrosine tRNA genes, respectively. 

**Table 3:** Frequency distribution of Mt-Cytochrome C oxidase variants in pompe and controls


Infant/Adult	Nucleotide	Locus	Amino acid change	R/N.R	Hm/Ht	Pompe	Control	P value
						+	+	

Adult	A7933G	Cyto co2	p.L116L	R	Hm	1	1	0.388
	C7939T	Cyto co2	p.F118F	R	Hm	1	0	0.219
	A8170G	Cyto co2	p.Gln195Gln	R	Hm	1	0	0.219
	T9581C	Cyto co3	p.Asn125Asn	R	Hm	1	0	0.219
	G9986A	Cyto co3	p.Gly260Gly	R	Hm	1	1	0.388
Infant	T7645C	MT co2	p.L20L	R	Hm	1	1	0.388
	G7805A	MT-CO2	p.Val74IL	R	Hm	1	0	0.219
	G7859A	MT-CO2	p.D92N	R	Hm	1	0	0.219
	T7861C	MT-CO2	p.D92D	R	Hm	1	1	0.388
	C7945T	MT-CO2	p.S120S	R	Hm	1	0	0.219
	C7990A	MT-CO2	p.L135L	R	Hm	1	0	0.219
	A8014T	MT-CO2	p.V143V	R	Hm	1	0	0.219
	C8137T	MT-CO2	p.F184F	R	Hm	1	2	0.520
	G8206A	MT CO2	p.Met207Met	R	Hm	1	3	0.990
	C9227G	Cyto co3	p.Ala7Ala	N.R	h.t	1	0	0.219
	A9336G	Cyt co3	p.Met44Val	R	Hm	1	0	0.219
	T9530C	Cyto co3	p.P108P	R	Hm	1	1	0.388
	C9776T	cyto co3	p.Asp190Asp	R	Hm	1	0	0.219
	T9949G	Cyto co3	p.Val248Gly	R	Hm	1	0	0.219
	T9963G	MT-Co3	p.Tyr253Asp	R	Hm	1	0	0.219
Both	G8269A	MT-CO2	p.X228X	R	Hm	3	0	0.219
	T9540C	cyto co3	p.L112L	R	Hm	3	0	0.219


R and N.R; Denote reported and not reported, respectively and Hm and Ht; Denotes homoplasmy and heteroplasmy, respectively.

## Discussion

PD is a heterogeneous neuromuscular disorder. Patients suffer from myopathy, hypotonia and other neuromuscular manifestations (5). Some tissues such as the nerve and muscle are more susceptible to mitochondrial dysfunction since these tissues are highly dependent on oxidative phosphorylation (10). According to previous studies, mitochondrial abnormality has been observed in PD patients (11-21). It is therefore possible that mitochondrial variants have a secondary role in PD. In this study, *MT-ATP6/ 8* and *Cytochrome C oxidase* genes of 28 PD patients were screened. The last complex in the mitochondria, *MT-ATP synthase*, plays an important role in the production of ATP. It has 14 subunits, of which 2, namely *MT-ATP6/8* are encoded by mtDNA (21). Variants in these genes may result in ATP production impairment in some vulnerable tissues such as the muscle (22).

The role of complex V variants has already been seen in the increase of free radicals. They can affect gene expression as a secondary factor. In these genes, some amino acids are conserved and any change could be potentially pathogenic (23, 24). Fourteen variants were found in *MT-ATP6/8* of which three were novel. One was a non-synonymous variant, however, it had a predicted benign effect on protein structure.

*MT-ATP6/8* expression decreased in the classic group and the number of variants in this group was more than the non-classic group. These are consistent with the severity of symptoms in the classic group. The missense variants A8502T, G9039A and G9055A replace Asparagine to Isoleucine, Methionine to Isoleucine and Alanine to Threonine respectively which all have a predicted damaging effect on protein structure. A8502T was reported by Gurses in 44 patients with Epilepsy (25). Asparagine is a polar amino acid which can form hydrogen bonds and acts as a neurotransmitter with Glutamate, while leucine is a non-polar and hydrophobic amino acid. This replacement may thus affect protein function as predicted by Polyphen 2. 

With respect to G8697A, methionine is a conserved amino acid. It has a sulfur in its structure that tends to form beta-sheets and despite owning hydrophobic properties, it can interact with some electrophilic regions. In contrast, isoleucine participates in alpha-helix structure and plays a role in ligand binding to protein. Such a replacement may change the structure and function of *MT-ATP6*. This variant was also observed in other studies (23, 26, 27). *MT-ATP6/8* genes were also studied in other neurodegenerative diseases such as Huntington (26), Friedreich’s ataxia and multiple sclerosis (MS) (28).

The C8684T variant in MT-ATP6 changes threonine to isoleucine. The former is a polar amino acid while the latter is non-polar. This change may affect the tertiary structure of the protein and its interaction with the ATP molecule. This variant was also observed in ataxia and autism (29). The C8684T was observed in MS and Huntington’s (23), and G8697A in MS (23) and ataxia telangiectasia (27). The C8562T is a synonymous variant which has been reported in patients with ataxia (29). A8456C and C8406T have benign effects. A8860G was found in 85.71% of patients. In addition, the variants G8697A and A8701G were previously reported in cardiomyopathy. This variant has also been reported in neurodegenerative diseases (30).

It is possible that the cardiomyopathy observed in PD patients is the result of the presence of these variants, by dysregulating *Mt.ATPase6/8* expression that was observed in this study. According to the previous study promoters are located in the D-loop region, this area is a hot spot therefor D-loop variants may change the sequence of promoters and binding affinity of transcription factors to enhancer or silencer elements (13, 17, 18). It seems that these variants along with other genetic and environmental factors are involved in development of PD. 

Mitochondrial DNA encodes 3 subunits for cytochrome C oxidase. It is the last enzyme in the mitochondrial respiratory chain and is responsible for electron transfer from the cytochrome to oxygen (31). Impairment of cytochrome C oxidase is clinically highly heterogeneous. It starts at any age and includes a diverse range of myopathy to severe multi-organ involvement (32).Genetic defects that affect the structure and function of this gene are usually severe and often lead to fatal metabolic disorders. Such disorders usually occur before the age of 2 and involve tissues such as the heart, muscle and liver, however, its manifestation in adulthood is with less severity. Severity of this disorder can vary even in family members. 

In the case of early onset cardiac muscle involvement is usually associated with hypertrophic cardiomyopathy, however, in late onset cases, myopathy and hypotonia are observed (33). It seems that the pattern of manifestations associated with *MT-cytochrome C oxidase* is similar to PD. Variants T9540C, T7645C and G8269A observed in this study were identical to those found in the study by Mkaouar-Rebai et al. (34) on patients with myopathy. These variants are likely to be related to myopathy and hypotonia symptoms of PD. The two variants T9949G and T9963G change valine to glycine and tyrosine to aspartic acid respectively. Polyphen 2 predicted these variants to have a damaging effect on protein structure. Both variants were in the classic group, thus suggesting that they may lead to more severe phenotypes in this group. 

The observed A8302G variant in lysine tRNA has been previously reported in encephalopathy (35). Also, Govindaraj et al. (36) reported this mutation in three patients with Madras motor neuron disease (MMND).

The variant T7572C located on the T arm of the gene encoding aspartate tRNA was previously reported by Reddy et al. (37) in myelodysplastic syndrome, however, Li et al. (38) found it as a neutral polymorphism, showing that this variant did not affect the secondary structure of the corresponding tRNA by using RNA fold.

G15928A, which is located at the anticodon stem of the tRNAThr, is shown to be a neutral polymorphism (38). The C15904T variant is also shown to be a polymorphism which occurs in the general population with low frequency (39). The C15904T and G15928A variants were observed in patients with encephalopathy by Houshmand et al. (40). Overall, validation of the function of each variant is recommended.

## Conclusion

The extent of mitochondrial involvement in the classic group was more severe than the non-classic group. According to these findings, it seems that mtDNA variants have a secondary role in PD. Understandingthe role of mitochondria in the pathogenesis of PD may potentially lead to the development of new therapeutic strategies. 
